# Remedies for Disorders of Stomach

**Published:** 1855-05

**Authors:** George Budd


### SOME REMEDIES FOR STOMACH DISORDERS.

BY GEORGE BUDD, M. D.

[In the 8th lecture on disorders of the stomach, Dr. Budd makes some useful remarks on the use of the mineral acids in various kinds of indigestion. But these have been so well treated of by Dr. Prout and others, that we will here only give Dr. Budd's remarks on *vegetable bitters.*]

Quinine, and the bitters generally, are especially grateful to persons who have injured their stomachs by hard drinking. With such persons they improve the appetite and strengthen digestion, and have a bracing effect upon the system at large.

In persons exhausted by over work, or wherever weakness of the stomach is the result of general debility from other causes, they often do much good in the same way—*by improving the appetite* and strengthening digestion.

They do harm in the organic diseases of the stomach; in plethoric states of the systen; and generally where there is a furred tongue, or where the urine throws down a sediment of lithic acid, or of lithate of ammonia. Their most striking effect is, to improve the appetite, when this has been impaired from hard drinking or from over work, or from nervous exhaustion from other causes; and the best time for giving them is from half an hour to an hour before meals.

The different bitters have not precisely the same effect. Columba has a sedative influence not possessed by others, and probably on this account has had a wider reputation as a remedy for mere indigestion. Gentian and chiretta (which is of the gentian tribe, and is much employed by practitioners in India) tend to increase the secretion of the liver, or, at any rate, do not impede the secretion of the liver, which quinine and quassia seem often to do. They are, therefore, better suited to bilious persons and to those cases of indigestion where the secretions of the liver are defective.

The different preparations of steel are especially useful in the indigestion that occurs in chlorosis, and generally where weakness of the stomach results from anæmia. They do harm in plethoric states of the system, and generally where there is furred tongue, or where the urine throws down a sediment of lithate of ammonia or of lithic acid.

The citrate, or ammonio-citrate, is the most agreeable preparation to the taste, and generally the most grateful to the stomach. If there be any disposition to sickness or nausea, or any tendency to furring of the tongue, it may be given in conjunction with bicarbonate of soda or potash. This makes a mixture having much the same effect as Griffith's mixture—the mistura ferri composita—and far more agreeable.

The muriated tincture of iron is more astringent than the other preparations, and may be given in conjunction with dilute muriatic acids, in the forms of indigestion suited to this latter medicine, when these exist in states of anæmia.

The sulphate of iron, like other metallic sulphates, has a tendency to cause sickness, and should not be given in cases where a disposition to sickness exists.

Steel medicines do good by improving the quality of the blood rather than by their immediate action on the coats of the stomach, and are best given at meal times. They then are mixed with food, and gradually absorbed with the products of digestion, and are less apt to offend the stomach and to cause head-ache than at other times.

Whenever steel medicines are given, it is essential that a regular action of the bowels be kept up. These medicines tend to confine the bowels and to cause evolution of sulphuretted hydrogen in them; and, unless this tendency be counteracted, they are apt to fur the tongue and cause head-ache.

The choice of purgatives is a very important matter in stomach disorders. The different purgatives exert their chief action on different portions of the intestinal canal; some excite the secretion or the peristaltic movement of one part, some of another. In disorders of the stomach and bowels, where a purgative is required, care should therefore be taken to select those which are least prone to irritate the injured or disordered part.

Castor oil, for example, offends the stomach, but acts very mildly on the large intestine. It should not be used in stomach disorders, or where, from any cause, a tendency to vomiting exists; but is better than any other purgative in dysentery, or during convalescence from typhoid fever, when the intestines are ulcerated, and in various other conditions where a speedy and sure purgative, and one not apt to irritate the large intestine, is required.

Senna acts chiefly on the small intestine, and, besides exciting its peristaltic action, increases the secretion from its mucous membrane. It acts, also, on the liver, increasing the secretion of bile. In conjunction with a few grains of calomel or blue pill, it is, as every one knows, one of the best purgatives in bilious states of the system, or where an evacuant is required; but in mere disorders of the stomach it is often objectionable, from the tendency it has to cause sickness.

The best purgatives in stomach disorders are aloes and colocynth, which exert their chief action on the large intestine. These medicines may do much harm when the large intestine is ulcerated or inflamed; but in simple ulcer of the the stomach, and in the most severe functional disorders of the stomach, they may generally be given without causing either pain of the stomach or sickness. In some kinds of functional disorder of the stomach, aloes seem, indeed, like other bitters, to improve the appetite and strengthen digestion.

Aloes appear to act more exclusively on the large intestine, and irritate the stomach less than colocynth, and hence, in stomach disorders is generally preferable to it.

Where, from the existence of piles, or from pregnancy, or some other condition, these medicines are objectionable, the best substitutes for them in stomach disorders are the saline purgatives, which exert their chief action on the small intestine, and have little tendency to cause pain in the stomach or sickness.—*Med. Times and Gazette.*

